# Image Fusion of CT and MR with Sparse Representation in NSST Domain

**DOI:** 10.1155/2017/9308745

**Published:** 2017-11-09

**Authors:** Chenhui Qiu, Yuanyuan Wang, Huan Zhang, Shunren Xia

**Affiliations:** Key Laboratory of Biomedical Engineering of Ministry of Education, Zhejiang University and the Zhejiang Provincial Key Laboratory of Cardio-Cerebral Vascular Detection Technology and Medicinal Effectiveness Appraisal, Hangzhou 310027, China

## Abstract

Multimodal image fusion techniques can integrate the information from different medical images to get an informative image that is more suitable for joint diagnosis, preoperative planning, intraoperative guidance, and interventional treatment. Fusing images of CT and different MR modalities are studied in this paper. Firstly, the CT and MR images are both transformed to nonsubsampled shearlet transform (NSST) domain. So the low-frequency components and high-frequency components are obtained. Then the high-frequency components are merged using the absolute-maximum rule, while the low-frequency components are merged by a sparse representation- (SR-) based approach. And the dynamic group sparsity recovery (DGSR) algorithm is proposed to improve the performance of the SR-based approach. Finally, the fused image is obtained by performing the inverse NSST on the merged components. The proposed fusion method is tested on a number of clinical CT and MR images and compared with several popular image fusion methods. The experimental results demonstrate that the proposed fusion method can provide better fusion results in terms of subjective quality and objective evaluation.

## 1. Introduction

Image fusion techniques combine multiple input images of the same scene or object to get a composite image that is expected to be more informative and suitable for human visual perception or further image processing compared to any of the input images [1–3]. Multimodal medical image fusion, one of the major image fusion applications, draws increasing attention from medical academia [4, 5] and has been used for joint diagnosis, preoperative planning, intraoperative guidance, interventional treatment, and so on [6, 7]. CT and MRI techniques have been widely used in clinical diagnosis and treatment; nevertheless the accurate differences between lesion, bone, and peritumoral soft tissue sometimes are hardly obtained by the single CT or MR images [7]. Although CT images provide electron density map required for accurate radiation dose estimation and superior cortical bone contrast, they are limited in soft tissue contrast. MRI provides good soft tissue contrast that permits better visualization of tumors or tissue abnormalities. However, it lacks signal from cortical bone and has image intensity values that have no relation to electron density [8]. Hence, for precise joint diagnoses and effective treatment procedures, the superior method for fusing CT and MR images should be explored urgently.

Multiscale transform (MST) theories are the most widely used tools in various image fusion situations [2, 3]. Classical MST tools include pyramid ones such as Laplacian pyramid (LP) [9] and gradient pyramid (GP) [10] and wavelet ones such as discrete wavelet transform (DWT) [11] and dual-tree complex wavelet transform (DTCWT) [12]. Since the pyramid and wavelet tools are originally proposed to process one-dimensional signals, even their improved versions cannot capture geometric structure information of the image very well. Current MST tools are multiscale geometric analysis (MGA) ones. MGA tools are able to extract geometric structure information including edges, curves, and textures of images much more effectively, since they are the “true” 2D transform. Curvelet transform (CVT) [13], contourlet transform (COT) [14], nonsubsampled contourlet transform (NSCT) [15], shearlet transform (ST) [16], and nonsubsampled shearlet transform (NSST) [17] are the most representative tools of MGA. CVT is proposed to represent a curve as the superposition of bases of various lengths and widths obeying a scaling law. NSCT is the shift-invariant version of COT essentially, which is built upon nonsubsampled multiscale pyramids and nonsubsampled directional filter banks. ST is much more efficient than COT in computational consumption. Besides, there are no restrictions on the number of directions and the size of support for the shearing [17]. NSST is the shift-invariant version of ST essentially.

The geometric structure information in different scales of the image can be extracted effectively by these MGA tools. The tools above are widely studied to fuse general multimodal images [8, 18–21].

Sparse representation (SR) is another popular and powerful theory used to solve the image fusion problem. SR addresses the signals' natural sparsity by simulating the sparse coding mechanism of human vision systems [22]. Yang and Li first apply SR theory to multifocus image fusion, and they use orthogonal matching pursuit (OMP) to implement sparse coding [23]. They also study the influence of dictionary and simultaneous OMP algorithm on the fusion of multimodal images [24].

Although MST-based and SR-based image fusion methods have achieved giant success, they have their special defects, respectively. We will discuss them in Section 4. In order to combine the advantages of MST and SR and avoid their defects, Wang et al. propose a fusion method that combines NSCT and SR, and OMP algorithm is used in their method to calculate the SR coefficients of the low-frequency components [25]. A general framework for image fusion combining MST (NSST is not included) and SR is proposed and discussed in detail by Liu et al. in [26]. They make comparisons and find that the method with combination of NSCT and SR which utilizes OMP algorithm for sparse coding obtains excellent fusion performance.

In this paper, we propose a novel fusion method to fuse clinical CT and MR images by combining NSST and SR. Firstly, the source images are both transformed to NSST domain. Then the high-frequency components are merged by the absolute-maximum rule, while the low-frequency components are merged with the SR-based approach. Wang et al. and Liu et al. utilize OMP algorithm to implement sparse coding, while the dynamic group sparsity recovery (DGSR) algorithm is first explored in our proposed method to improve the performance of SR-based approach. Finally, the fused image is obtained by performing the inverse NSST on the merged low-frequency and high-frequency components.

Our proposed fusion method is tested on forty pairs of clinical brain CT and MR anatomical images and compared with seven popular image fusion methods. MR imaging techniques can provide four kinds of anatomical images which are all considered in our research. The experimental results demonstrate that the proposed fusion method can provide superior fusion results in terms of subjective quality and objective evaluation.

The remainder of this paper is organized as follows. The related work including NSST and SR is presented in Section 2. The proposed fusion method is described in Section 3. Experiments and discussions are shown in Section 4. Finally, we draw the conclusions and depict some future work in Section 5.

## 2. Related Work

### 2.1. Nonsubsampled Shearlet Transform (NSST)

Shearlet has been regarded as the best sparse directional image representation frame among MST theories so far [5]. In dimension *n* = 2, the affine systems with composite dilations are described as follows:

(1) $$\begin{array}{c} \psi_{j,l,k}\left(\;x\;\right)={\left|\;\det A\;\right|}^{j/2}\psi \left(\;S^{l}A^{j}x-k\;\right), \end{array}$$

where *ψ* ∈ *L*^2^(*ℝ*^2^), *A* refers to anisotropy matrix which is associated with scale transformations, and *S* refers to shear matrix which is associated with area-preserving geometrical transformations, such as rotations and shear. *j*, *l*, and *k* are scale, direction, and shift parameter, respectively. For each *a* > 0 and *s* ∈ *ℝ*, the matrices of *A* and *S* which play vitally important roles in the process of ST are given as follows [17]:

(2) A=a00a,S=1s01.

Assume that *a* = 4 and *s* = 1; then we obtain

(3) A0=4002,S0=1101.

Let $A_{1}=\left[\begin{array}{cc} 2 & 0\\ 0 & 4 \end{array}\right]$ and $S_{1}=\left[\begin{array}{cc} 1 & 0\\ 1 & 1 \end{array}\right]$, for any *ξ* = (*ξ*_1_, *ξ*_2_) ∈ *ℝ*^2^ and *ξ*_1_ ≠ 0; also let *ψ*^(0)^ and *ψ*^(1)^ be given by

(4) ψ^0ξ=ψ^0ξ1,ξ2=ψ^1ξ1ψ^2ξ2ξ1ψ^1ξ=ψ^1ξ1,ξ2=ψ^1ξ2ψ^2ξ1ξ2,

where ${\hat{\psi }}_{1},{\hat{\psi }}_{2}\in C^{\infty }(\hat{\mathbb{R}})$, $supp\;{\hat{\psi }}_{1}\subset \left[-1/2,-1/16\right]\cup \left[1/16,1/2\right]$, and $supp\;{\hat{\psi }}_{2}\subset \left[-1,1\right]$.

Each element of ${\hat{\psi }}_{j,l,k}$ is supported on a pair of trapezoids, of approximate size 2^*j*^ × 2^2*j*^, oriented along lines of slope *l*2^−*j*^.

Then the ST function is obtained:

(5) ψj,l,k0x=23j/2ψ0S0lA0jx−kψj,l,k1x=23j/2ψ1S1lA1jx−k,

where *j* ≥ 0, −2^*j*^ ≤ *l* ≤ 2^*j*^ − 1, and *k* ∈ *ℤ*^2^.

ST has the following properties: well spatial and frequent localization, strong selectivity of anisotropic directionality, well parabolic scaling, and approximately sparse representation. However, ST engenders the Gibbs phenomena because of the lack of shift invariance. NSST is the shift-invariant version of ST. It utilizes nonsubsampled Laplacian pyramid (NSLP) filters as the substitutes for the LP filters which are used in the ST mechanism. Multiscale decomposition is implemented by NSLP. One low-frequency component and one high-frequency component can be produced at each NSLP decomposition level. And the next NSLP decomposition is performed on the last (previous) low-frequency component to capture the singularities of the source image iteratively. When the decomposition level is set to *J*, the source image is decomposed into *J* + 1 components with the same size of the source image, in which one is the low-frequency component.

**Figure 1:**
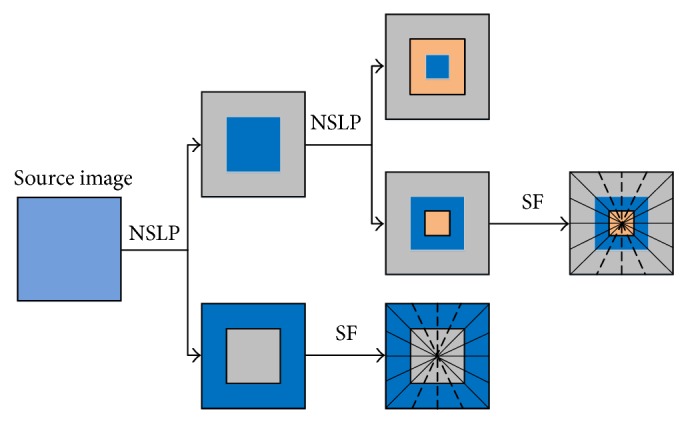
Two-level multiscale and multidirectional decompositions of the NSST.

Shearing filter (SF) is performed on high-frequency components of each NSLP decomposition level without subsampling to achieve the multidirectional factorization, which ensures the shift invariance property of NSST. Assume that SF performs the directional decomposition with *l* stages on a high-frequency component decomposed by NSLP; then 2^*l*^ directional subbands with the same size as the source image are produced [17]. Two-level decomposition of the NSST is shown in Figure 1. This schematic diagram illustrates the NLSP decomposition and the corresponding directional decomposition by SF.

### 2.2. Sparse Representation (SR)

SR theory is based on the assumption that the natural signals can be represented or approximately represented by a linear combination of a “few” atoms from dictionary [22]. For signals Γ ⊂ *ℝ*^*n*^, SR theory indicates the existence of a dictionary **D** ∈ *ℝ*^*n*×*m*^, which contains *m* prototype signals that are referred to atoms. Then, for any signal **x** ∈ Γ, there is a linear combination of atoms from **D** which can approximate to **x** very well. The signal **x** can be expressed as **x** ≈ **Dα**, where **α** ∈ *ℝ*^*m*^ is the unknown sparse coefficient vector. It usually assumes that *n* < *m*, which implies that **D** is the overcompleted and redundant dictionary. So there are numerous feasible solutions of the underdetermined system **x** ≈ **Dα**. Finding the sparsest **α** that contains the fewest nonzero entries involves solving the following optimization problem:

(6) arg minα α0s.t. Dα−x2<ε,

where ‖**α**‖_0_ denotes the number of nonzero entries in **α**; *ε* > 0 is an error tolerance.

The above optimization is an NP-hard problem. The main idea to solve this problem is obtaining an approximate solution instead of the sparsest one. OMP is the representative algorithm to get the approximate solution.

## 3. Proposed Fusion Method

**Figure 2:**
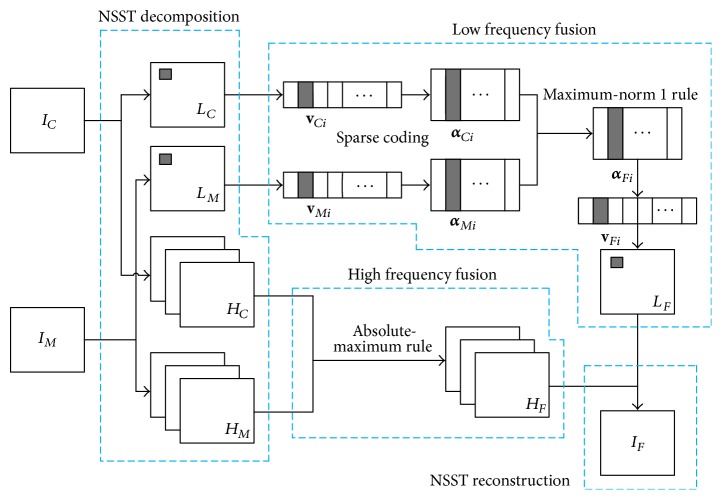
The schematic diagram of the proposed method.

The proposed fusion method of CT and MR images mainly contains four phases: NSST decomposition, high-frequency fusion, low-frequency fusion, and NSST reconstruction. The schematic diagram of the proposed method is shown in Figure 2.

### 3.1. NSST Decomposition

NSST decomposition is performed on CT and MR images {*I*_*C*_, *I*_*M*_} to obtain high-frequency components that are denoted as {*H*_*C*_, *H*_*M*_} and low-frequency components that are denoted as {*L*_*C*_, *L*_*M*_}.

### 3.2. High-Frequency Fusion

The high-frequency components at different levels contain much salient information of regional boundary, edge, and line features. The larger absolute values of the coefficients in each of the high-frequency components indicate more important salient information. Therefore, the popular absolute-maximum rule is appropriate to merge the high-frequency components {*H*_*C*_, *H*_*M*_}. The coefficients of high-frequency components *H*_*C*_ and *H*_*M*_ and the fused image *H*_*F*_ are denoted as *h*_*C*_ and *h*_*M*_ and *h*_*F*_, respectively. The rule is expressed as follows:

(7) $$\begin{array}{c} h_{F}^{}=\left\{\begin{array}{cc} h_{C}^{} & if\;\;\left|h_{C}^{}\right|\geq \left|h_{M}^{}\right|\\ h_{M}^{} & otherwise. \end{array}\right. \end{array}$$

### 3.3. Low-Frequency Fusion

*L*_*C*_ and *L*_*M*_ are merged with SR-based fusion approach in our research. Image fusion methods always depend on the local information of source images. Besides, the stability and efficiency of sparse coding should be taken into account. Hence, the source images need to be divided into image patches.

The sliding window technique is adopted to divide *L*_*C*_ and *L*_*M*_ into image patches of size *d* × *d* from upper left to lower with a suitable step length, respectively [23]. Then all patches of *L*_*C*_ are transformed into vectors via lexicographic ordering and normalization operation. And all these vectors constitute the matrix **V**_*C*_, in which each column corresponds to one patch of *L*_*C*_. The size of **V**_*C*_ is (*d* · *d*)×((*M* − *d* + 1)·(*N* − *d* + 1)). **V**_*M*_ can be obtained in the same way.

For the *i*th column vector **v**_*Ci*_ of **V**_*C*_, its sparse representation is calculated by the DGSR algorithm. According to (6), the sparse coding vector **α**_*Ci*_ for **v**_*Ci*_ is obtained as follows:

(8) αCi=arg minα α0s.t. Dα−vCi2<ε.

Similarly, we can get the sparse coding vector **α**_*Mi*_ for **v**_*Mi*_ which is the *i*th column vector of **V**_*M*_.

(9) αMi=arg minα α0s.t. Dα−vMi2<ε,

where **D** is the offline learned dictionary that is trained by *K*-means singular value decomposition (*K*-SVD) algorithm [27].

Then **α**_*Ci*_ and **α**_*Mi*_ are merged by maximum-norm 1 rule to produce the fused sparse vectors **α**_*Fi*_. The fusion rule is expressed as follows:

(10) $$\begin{array}{c} \alpha_{Fi}=\left\{\begin{array}{cc} \alpha_{Ci} & if\;\;{\left\|\alpha_{Ci}\right\|}_{1}\geq {\left\|\alpha_{Mi}\right\|}_{1}\\ \alpha_{Mi} & otherwise. \end{array}\right. \end{array}$$

The *i*th column vector **v**_*Fi*_ of the matrix **V**_*F*_ is calculated by

(11) $$\begin{array}{c} v_{Fi}=Da_{Fi}. \end{array}$$

Then **V**_*F*_ can be constituted by all these column vectors.

Finally, the low-frequency component *L*_*F*_ of the fused image *I*_*F*_ is reconstructed by **V**_*F*_. This reconstruction is the inverse process of normalization and sliding window technique.

In the fusion application of CT and MR images, the nonzero sparse coefficients of source images are not randomly distributed but are group-clustered. They tend to be clustered into groups, although the clustering group structures are dynamic and unpredictable. Therefore, DGSR algorithm that prunes data residues in the iterative process according to both sparsity and the group clustering trend is appropriate for our research. The experimental results in [28] demonstrate that the DGSR algorithm achieves better performance than OMP algorithm. In this paper, the main work is to explore the image fusion method for CT and MR; the details of DGSR algorithm can be found in [28]. The MATLAB codes of DGSR algorithm and relevant experiments can be downloaded at http://ranger.uta.edu/~huang/Downloads.htm.

### 3.4. NSST Reconstruction

The inverse NSST is performed on both low frequency *L*_*F*_ and high frequency *H*_*F*_ to reconstruct the final fused image *I*_*F*_.

## 4. Experiments and Discussions

### 4.1. CT and MR Images

CT imaging techniques provide anatomical images of tissues or lesions. MR-T1W, MR-T2W, and MR-PDW are derived from different settings of longitudinal relaxation time T1, transversal relaxation time T2, echo pulse sequence, and proton density of tissues. T1W means that the contrasts of tissues in the MR image mainly depend on T1 weight. T2W and PDW are of the similar meaning. MR-CE image is acquired by adding gadolinium-containing contrast medium, which is mainly used for the definite diagnosis of cerebral tumor. They are all MR anatomical images.

**Figure 3:**
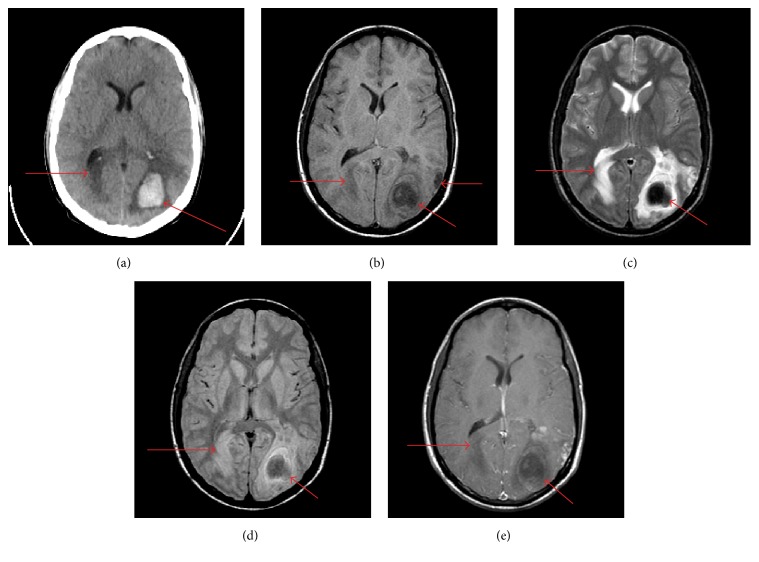
The clinical brain CT and MR images. (a) CT image, (b) MR-T1W image, (c) MR-T2W image, (d) MR-PDW image, and (e) MR-CE image.

Because of the limited space of paper, only one representative group of clinical brain CT and MR images are presented as the source images which are shown in Figure 3. The images of Figure 3 are the 12th slices of a Ewing's sarcoma patient at the age of 22. He had a right homonomous hemianopia, a left inferior quadrantanopia, right lower extremity hyperreflexia, and right extensor plantar response. The images are of the same slice of the same patient so that we can find the difference of different imaging modalities obviously. They can be downloaded from http://www.med.harvard.edu/aanlib/.

### 4.2. Experimental Setting

The clinical brain CT and MR images above have the same size, 256 × 256, and they are preregistered in pairs. All the experiments are implemented in MATLAB 7.14 and on a PC with 2.66-GB CPU, 4 GB RAM, and 32-bit OS.

To assess the performance of the proposed method, the GP [10], DTCWT [12], CVT [18], NSCT [19], NSST [20], SR-OMP [23], and NSCT-SR-OMP [25, 26] based fusion methods are implemented for comparison.

The fusion rules for low-frequency components of these MST-based methods are all chosen as “averaging” rule. The fusion rules for high-frequency components of these MST-based methods are all chosen as “max-absolute” rule [2]. For SR-OMP based method, the fusion rule is chosen as “max-norm 1” rule [23]. For NSCT-SR-OMP based method, the fusion rules for low-frequency and high-frequency components are the same as the proposed method's fusion rules [25, 26]. For these MST-based methods, NSCT-SR-OMP based method, and the proposed method, the decomposition levels are all set to 4, which are most appropriate according to our experiments and the analysis in [2, 26]. For DTCWT-based method, the filters of first level and other levels are selected as “5-3” and “q_a,” respectively. For NSCT-based and NSCT-SR-OMP based methods, the pyramidal filters and directional filters are selected as “pyrexc” and “7-9,” respectively. And the directional numbers form coarser to finer scales are chosen as 2^3^, 2^3^, 2^4^, and 2^4^, respectively. For NSST-based method and the proposed method, NSLP filters are selected as “pyrexc.” The directional numbers are the same as aforementioned. And the Meyer wavelet window is used for SF. The sizes of SFs which are ordered from coarser to finer scales are chosen as 32, 32, 16, and 16, respectively [17]. For SR-OMP and NSCT-SR-OMP based methods and the proposed method, the size of image patches is set to 8 × 8, the step length is set to 2, the error tolerance is set to 0.1, and the dictionary size is set to 64 × 256 [2, 23].

### 4.3. Objective Evaluation Metrics

Generally, image fusion results can be evaluated in subjective and objective ways. Subjective ways are difficult to execute because they are based on psychovisual testing and cannot be applied in an automatic system. Besides, there is little visual difference among the fusion results in most cases, so subjective ways are difficult to correctly evaluate the fusion results. Therefore, many objective evaluation metrics have been developed [2, 3]. In this paper, standard deviation (STD) [29], mean structural similarity (M-SSIM) [30], mutual information (MI) [31], sum of the correlations of differences (SCD) [32], *Q*_0_ [33], *Q*_*W*_ [34], *Q*_*E*_ [34], *Q*^*AB*/*F*^ [35], and visual information fidelity for fusion (VIFF) [36] are used to evaluate the fusion results objectively. The larger values of these metrics imply the better fusion results. It is worthwhile to note that if the fused images are used for preoperative planning, intraoperative guidance, and interventional treatment, which depend on further image processing, the superiority of objective evaluation is important and valuable. We also give simply subjective assessments on the fusion results.

### 4.4. Results and Discussions

**Figure 4:**
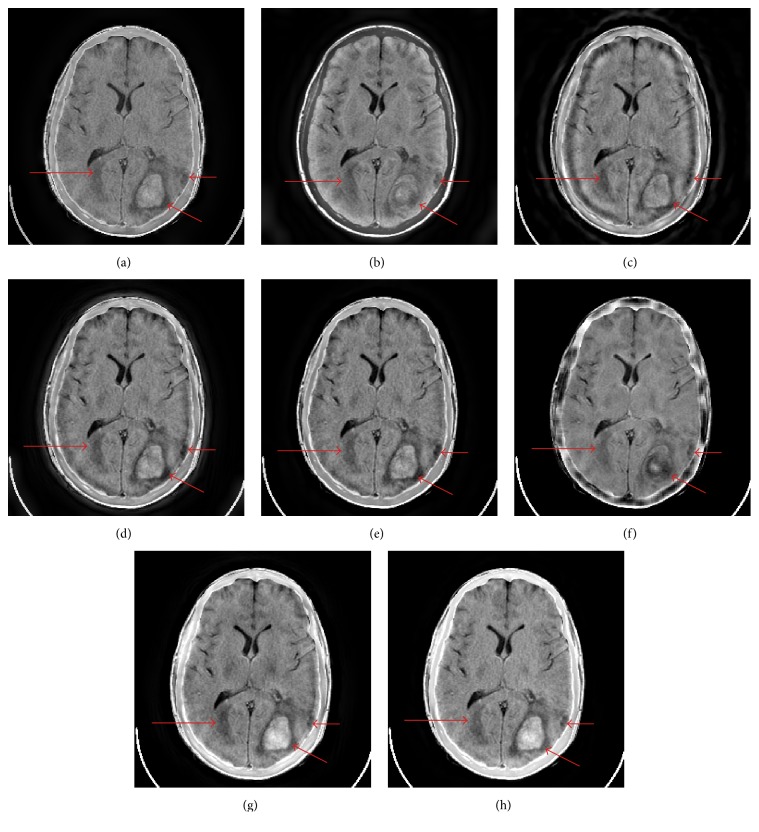
The fusion results of Figures 3(a) and 3(b). ((a)–(h)) GP, DTCWT, CVT, NSCT, NSST, SR-OMP, NSCT-SR-OMP, and proposed method.

Figure 4 shows the fusion results of Figures 3(a) and 3(b) with different fusion methods. In the right halves of Figure 3(b) and Figures 4(a)–4(h), there are two lesions labeled by the arrows. Actually they are the one lesion. And the small one is the diffusion of the large one. The small lesion in right half and the lesion in left half of Figures 4(a), 4(b), 4(c), and 4(f) is blurred to some extent. Although Figures 4(d) and 4(e) exhibit the small lesion in right half well, they cannot exhibit the lesion in left half with good visual quality. The cerebral bony structure is the basic sclerous tissue in brain anatomical image. Only Figures 4(g) and 4(h) show the cerebral bony structure as well as the expected effect. On the whole, the visual information of tissues and lesions of CT and MR-T1W images is most perfectly preserved in Figures 4(g) and 4(h). Besides, the CVT- and NSCT-based methods produce artifacts along the cerebral bony structure. Although SR-OMP based method can capture the salient information, it cannot extract the blurry features (such as the lesion in left half) effectively.

Table 1 gives the objective evaluation metrics of the fusion results of Figures 3(a) and 3(b) with different fusion methods. The best result for each metric is labeled in bold. The second best result for each metric is labeled with italic font. The proposed method provides the best results for six metrics and the second best result for one metric. Although the fusion results of NSCT-SR-OMP based method and the proposed method are very similar in subjective quality, the proposed method is superior to NSCT-SR-OMP based method in all of the objective evaluation metrics. In addition, the computational efficiency of the proposed method is much higher than that of NSCT-SR-OMP based method because the decomposition and reconstruction of NSST are much faster than those of NSCT [17].

**Figure 5:**
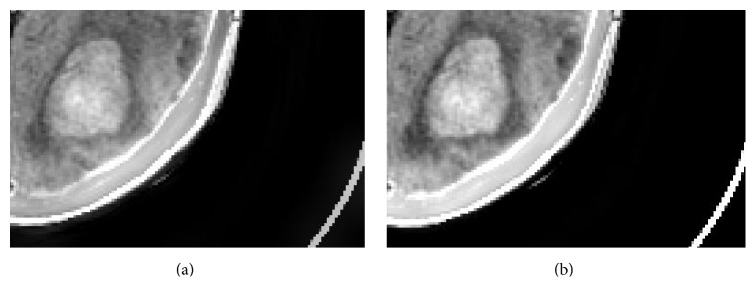
The zoomed-in version of the bottom-right part of Figures 4(g) and 4(h): (a) the zoomed-in version of Figure 4(g); (b) the zoomed-in version of Figure 4(h).

The zoomed-in version of the bottom-right part of Figures 4(g) and 4(h) is shown in Figure 5. We can find that the contrast of the larger lesion and its limbic region in Figure 5(b) is higher than the corresponding part in Figure 5(a). This verdict coincides with the STD metric evaluation. Besides, the visual quality of Figure 5(b) is higher than that of Figure 5(a), which is consistent with the VIFF metric. (Note that medical display system provides better visual effect than common display system; it distinguishes the difference between Figures 5(a) and 5(b) more clearly.)

**Figure 6:**
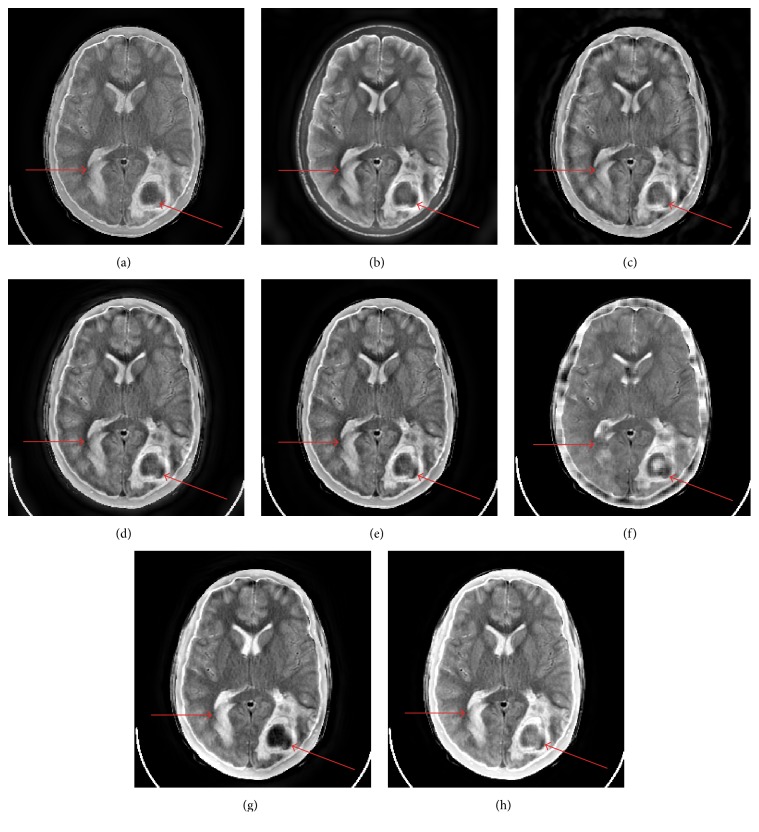
The fusion results of Figures 3(a) and 3(c). ((a)–(h)) GP, DTCWT, CVT, NSCT, NSST, SR-OMP, NSCT-SR-OMP, and proposed method.

Figure 6 shows the fusion results of Figures 3(a) and 3(c) with different fusion methods. The subjective analysis of Figure 6 is similar to the subjective assessment of Figure 4. The proposed method provides the best visual quality of the lesion labeled by the arrow, sulcus, and bony structure. NSCT-SR-OMP based method provides similar but little worse subjective visual quality with careful inspection.

Table 2 gives the objective evaluation metrics of the fusion results of Figures 3(a) and 3(c) with different fusion methods. The proposed method provides the best results for six metrics and the second best result for one metric.

**Figure 7:**
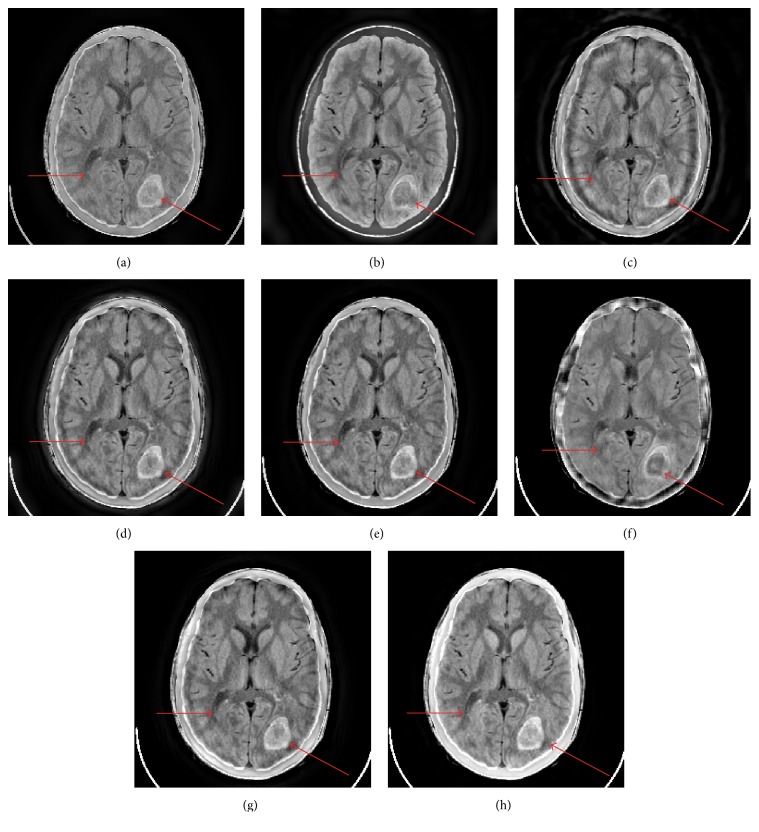
The fusion results of Figures 3(a) and 3(d). ((a)–(h)) GP, DTCWT, CVT, NSCT, NSST, SR-OMP, NSCT-SR-OMP, and proposed method.

**Figure 8:**
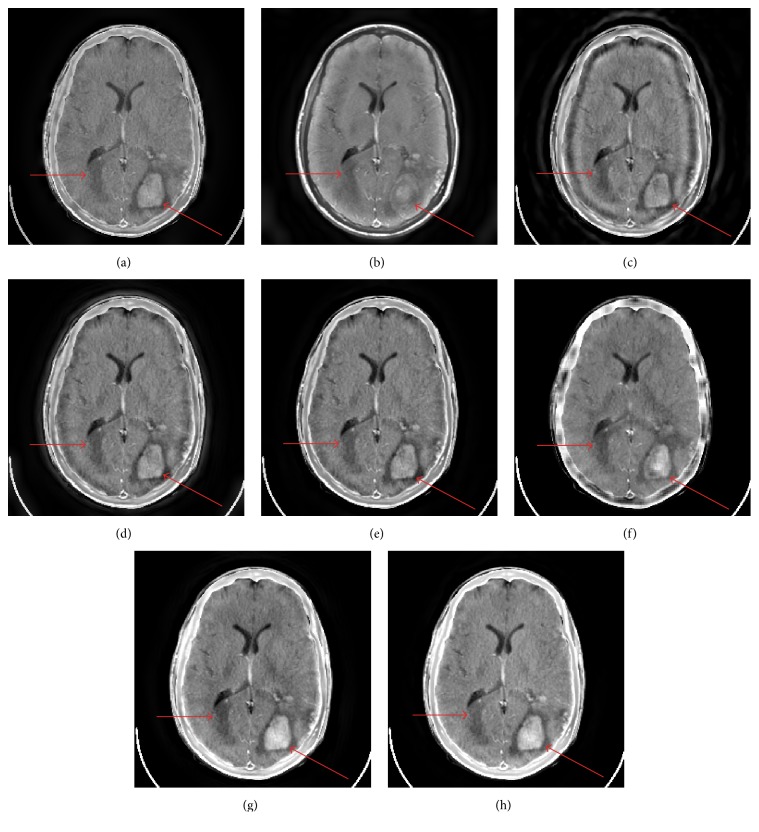
The fusion results of Figures 3(a) and 3(e). ((a)–(h)) GP, DTCWT, CVT, NSCT, NSST, SR-OMP, NSCT-SR-OMP, and proposed method.

Figures 7 and 8 show the fusion results of Figures 3(a) and 3(d) and Figures 3(a) and 3(e) with different fusion methods, respectively. The proposed method provides the best visual fusion performances in the two cases.

Tables 3 and 4 give the objective evaluation metrics of the fusion results of Figures 3(a) and 3(d) and Figures 3(a) and 3(e) with different fusion methods, respectively. The proposed method provides the best results for six metrics and the second best result for one metric in Table 3. It also provides the best results for five metrics and the second best result for one metric in Table 4.

Table 5 gives the objective evaluation metrics of the fusion results of forty pairs of clinical brain CT and different MR modalities with different fusion methods. The means and the standard deviations of different metrics of different methods are shown in Table 5. We can find that the proposed method provides the best results for six metrics.

NSST is the most representative MST tool, which can extract the geometric structure information of source images more effectively than NSCT, CVT, and classical MST tools. The salient information such as edges, curves, and textures can be captured by NSST very well and can be preserved in its high-frequency components. Therefore, NSST-based method obtains the best results of *Q*_*E*_ or *Q*^*AB*/*F*^ metrics in some cases. However, NSST cannot express the low-frequency components sparsely. If ordinary fusion rule is utilized to merge the low-frequency components, the fusion performance may be degraded as the low-frequency components contain a lot of energy of source images.

SR theory can represent an image in the approximately sparsest sense. It is the best approach of two-dimensional signal in terms of information theory, so SR-based method obtains the best results of MI metric form Tables 1–5. While SR-based fusion approach can represent the underlying salient information of source images very efficiently, it is difficult to reconstruct the small-scale details of source images and the blurred features.

The proposed method combines NSST and SR in order to preserve their advantages and avoid their defects. DGSR algorithm is first explored in the proposed method to ensure the satisfactory performance when SR-based fusion approach is implemented on low-frequency components. It is worthwhile to note that NSST plays a more important role than SR in the proposed fusion method, since the fusion framework is mainly based on NSST. The geometric structure information including edges, curves, and textures of images is preserved in high-frequency components of NSST, while a minority of salient features and a majority of energy are preserved in the low-frequency components of NSST. The SR-based approach is used to fuse the low-frequency components and DGSR algorithm is explored to calculate sparse representation coefficients of the low-frequency components. We can find that the proposed method has the superiority in STD, M-SSIM, SCD, *Q*_0_, *Q*_*W*_, and VIFF metrics from the tables.

## 5. Conclusions and Future Work

In this paper, we have proposed a novel image fusion method for fusing CT and MR images, which combines the advantages of NSST and SR. Moreover, DGSR algorithm is utilized when the SR-based fusion approach is performed on the low-frequency components of NSST decompositions. To assess the performance of the proposed method, the GP, DTCWT, CVT, NSCT, NSST, SR-OMP, and NSCT-SR-OMP based fusion methods are implemented for comparison. The experimental results show that the proposed method provides superior fusion results in terms of subjective quality and objective evaluation metrics.

However, fusing MR and low-dose CT images is not considered in our paper because the low-dose CT image contains a lot of noise, which is complicated and cannot be modeled as Gaussian distribution or Rician distribution. We may concentrate on this fusion topic in the future.

Besides, the automatic or guided target delineation in radiotherapy planning which is based on the fused CT and MR images will be studied in the future.

## Figures and Tables

**Table 1:** Objective evaluation metrics of the fusion results of Figures 3(a) and 3(b).

Method	STD	M-SSIM	MI	SCD	*Q*_0_	*Q*_*W*_	*Q*_*E*_	*Q* ^*AB*/*F*^	VIFF
Proposed	**83.1426**	**0.7511**	*2.7804*	**1.4081**	**0.3614**	**0.6502**	0.4119	0.4911	**0.4494**
NSCT-SR-OMP	78.2445	0.6689	2.6225	1.1685	0.3478	0.6320	0.4133	0.4765	0.4124
SR-OMP	67.3711	0.7392	**3.1058**	0.8483	0.3164	0.5886	0.4131	0.5091	0.2517
NSST	69.2338	0.6853	2.5469	1.0222	0.3535	0.6504	**0.4364**	0.5205	0.3862
NSCT	66.1168	0.5354	2.3739	0.8571	0.3266	0.5942	0.3935	0.4852	0.3302
CVT	67.1317	0.5582	2.2612	0.8859	0.3010	0.6186	0.4017	0.4629	0.3319
DTCWT	64.1289	0.5115	2.6224	0.8886	0.2430	0.3381	0.2401	**0.5746**	0.1849
GP	66.3231	0.6104	2.5530	0.9997	0.3505	0.6201	0.4042	0.4868	0.3440

**Table 2:** Objective evaluation metrics of the fusion results of Figures 3(a) and 3(c).

Method	STD	M-SSIM	MI	SCD	*Q*_0_	*Q*_*W*_	*Q*_*E*_	*Q* ^*AB*/*F*^	VIFF
Proposed	**85.4593**	**0.7386**	*2.9224*	**1.4621**	**0.3824**	**0.7690**	0.4827	0.5113	**0.4131**
NSCT-SR-OMP	76.8654	0.6591	2.8395	1.1346	0.3718	0.7587	**0.5001**	0.5217	0.3669
SR-OMP	73.0928	0.7235	**3.1352**	1.0630	0.3353	0.7170	0.4729	0.5192	0.2911
NSST	69.3967	0.6643	2.6868	1.0580	0.3670	0.7372	0.4851	**0.5332**	0.3338
NSCT	66.7627	0.5297	2.6151	0.9171	0.3433	0.6916	0.4680	0.4921	0.2897
CVT	67.2029	0.5603	2.4766	0.9319	0.3152	0.7006	0.4542	0.4715	0.2837
DTCWT	61.8421	0.4939	2.5247	0.8863	0.2558	0.2433	0.1876	0.4946	0.1420
GP	66.1162	0.6455	2.7886	1.0829	0.3662	0.6868	0.4535	0.4983	0.3151

**Table 3:** Objective evaluation metrics of the fusion results of Figures 3(a) and 3(d).

Method	STD	M-SSIM	MI	SCD	*Q*_0_	*Q*_*W*_	*Q*_*E*_	*Q* ^*AB*/*F*^	VIFF
Proposed	**83.5111**	**0.7583**	*2.7913*	**1.3901**	**0.3749**	**0.6762**	0.4247	0.4896	**0.4487**
NSCT-SR-OMP	74.3309	0.6554	2.5727	1.0432	0.3611	0.6548	0.4312	0.4820	0.3996
SR-OMP	68.0421	0.7522	**2.8751**	0.8643	0.3365	0.6083	0.4174	0.5042	0.2662
NSST	69.1365	0.6885	2.5367	1.0001	0.3679	0.6619	**0.4470**	0.5147	0.3804
NSCT	66.1375	0.5378	2.3410	0.8138	0.3371	0.5891	0.3997	0.4491	0.3288
CVT	67.2095	0.5739	2.2814	0.8713	0.3167	0.6222	0.4038	0.4503	0.3214
DTCWT	63.6317	0.5042	2.6157	0.8570	0.2564	0.2516	0.1870	**0.5476**	0.1790
GP	66.7812	0.6558	2.5425	0.9912	0.3638	0.6216	0.4109	0.4737	0.3361

**Table 4:** Objective evaluation metrics of the fusion results of Figures 3(a) and 3(e).

Method	STD	M-SSIM	MI	SCD	*Q*_0_	*Q*_*W*_	*Q*_*E*_	*Q* ^*AB*/*F*^	VIFF
Proposed	**82.3036**	*0.7453*	2.9034	**1.3673**	**0.3829**	**0.6598**	0.3877	0.5277	**0.4598**
NSCT-SR-OMP	78.7649	0.6805	2.9842	1.1264	0.3798	0.6478	0.3888	0.5109	0.4472
SR-OMP	72.5496	**0.7533**	**3.4771**	0.7848	0.3568	0.6178	0.4028	0.5348	0.3257
NSST	70.0334	0.6944	2.6821	0.9589	0.3725	0.6573	**0.4181**	**0.5514**	0.4028
NSCT	66.9852	0.5503	2.5579	0.7918	0.3492	0.6115	0.3820	0.5033	0.3480
CVT	68.0393	0.5602	2.3974	0.8071	0.3157	0.6202	0.3600	0.4694	0.3529
DTCWT	64.8234	0.5444	2.7009	0.8209	0.2000	0.3193	0.2279	0.5390	0.1816
GP	66.4735	0.6117	2.6955	0.9055	0.3758	0.6300	0.4058	0.5206	0.3567

**Table 5:** Objective evaluation metrics of the fusion results of the forty pairs of clinical brain CT and MR images (mean/STD).

Method	STD	M-SSIM	MI	SCD	*Q*_0_	*Q*_*W*_	*Q*_*E*_	*Q* ^*AB*/*F*^	VIFF
Proposed	**83.5748/1.5512**	**0.7523/0.0245**	2.9104/0.1727	**1.4430/0.1321**	**0.3798/0.0284**	**0.7166/0.0403**	0.4495/0.0464	0.5037/0.0384	**0.4874/0.0778**
NSCT-SR-OMP	77.1774/2.3899	0.6775/0.0275	2.7336/0.1759	1.1635/0.1341	0.3605/0.0195	0.7117/0.0419	0.4547/0.0385	0.5074/0.0298	0.4432/0.0802
SR-OMP	71.4563/3.1108	0.7381/0.0228	**3.1898/0.2426**	0.9630/0.1418	0.3414/0.0248	0.6827/0.0446	0.4481/0.0372	0.5284/0.0302	0.3224/0.0584
NSST	69.3828/1.9412	0.6874/0.0245	2.6168/0.1203	1.0590/0.1133	0.3601/0.0179	0.7043/0.0304	**0.4612/0.0305**	**0.5378/0.0293**	0.4068/0.0704
NSCT	66.7117/2.0838	0.5470/0.0273	2.4958/0.1426	0.9087/0.1121	0.3363/0.0166	0.6593/0.0362	0.4283/0.0276	0.4850/0.0260	0.3555/0.0681
CVT	67.2008/1.9224	0.5680/0.0230	2.3954/0.1572	0.9241/0.1112	0.3119/0.0162	0.6668/0.0316	0.4176/0.0306	0.4649/0.0239	0.3494/0.0687
DTCWT	62.7511/2.5960	0.5185/0.0331	2.5262/0.1377	0.8533/0.1033	0.2360/0.0280	0.3276/0.0654	0.2409/0.0489	0.5097/0.0383	0.1888/0.0441
GP	65.6848/1.7288	0.6384/0.0271	2.6640/0.1081	1.0159/0.1336	0.3576/0.0174	0.6686/0.0261	0.4413/0.0302	0.5050/0.0325	0.3653/0.0497

## References

[1] Goshtasby A. A., Nikolov S. (2007). Image fusion: advances in the state of the art. *Information Fusion*.

[2] Li S., Yang B., Hu J. (2011). Performance comparison of different multi-resolution transforms for image fusion. *Information Fusion*.

[3] Li S., Kang X., Fang L., Hu J., Yin H. (2017). Pixel-level image fusion: a survey of the state of the art. *Information Fusion*.

[4] James A. P., Dasarathy B. V. (2014). Medical image fusion: a survey of the state of the art. *Information Fusion*.

[5] Du J., Li W., Lu K., Xiao B. (2016). An overview of multi-modal medical image fusion. *Neurocomputing*.

[6] Webster G. J., Kilgallon J. E., Ho K. F., Rowbottom C. G., Slevin N. J., Mackay R. I. (2009). A novel imaging technique for fusion of high-quality immobilised MR images of the head and neck with CT scans for radiotherapy target delineation. *British Journal of Radiology*.

[7] Dai J., Wang X., Dong Y., Yu H., Yang D., Shen G. (2012). Two- and three-dimensional models for the visualization of jaw tumors based on CT-MRI image fusion. *The Journal of Craniofacial Surgery*.

[8] Ganasala P., Kumar V. (2014). CT and MR image fusion scheme in nonsubsampled contourlet transform domain. *Journal of Digital Imaging*.

[9] Burt P. J., Adelson E. H. (1983). The laplacian pyramid as a compact image code. *IEEE Transactions on Communications*.

[10] Petrović V. S., Xydeas C. S. (2004). Gradient-based multiresolution image fusion. *IEEE Transactions on Image Processing*.

[11] Li H., Manjunath B. S., Mitra S. K. (1995). Multisensor image fusion using the wavelet transform. *CVGIP: Graphical Models and Image Processing*.

[12] Lewis J. J., O'Callaghan R. J., Nikolov S. G., Bull D. R., Canagarajah N. (2007). Pixel- and region-based image fusion with complex wavelets. *Information Fusion*.

[13] Candès E. J., Donoho D. L. (2001). Curvelets and curvilinear integrals. *Journal of Approximation Theory*.

[14] Do M. N., Vetterli M. (2005). The contourlet transform: an efficient directional multiresolution image representation. *IEEE Transactions on Image Processing*.

[15] da Cunha A. L., Zhou J., Do M. N. (2006). The nonsubsampled contourlet transform: theory, design, and applications. *IEEE Transactions on Image Processing*.

[16] Labate D., Lim W.-Q., Kutyniok G., Weiss G. Sparse multidimensional representation using shearlets.

[17] Easley G., Labate D., Lim W.-Q. (2008). Sparse directional image representations using the discrete shearlet transform. *Applied and Computational Harmonic Analysis *.

[18] Mehta S., Marakarkandy B. (2013). CT and MRI image fusion using curvelet transform. *Journal of Information, Knowledge and Research in Electronics and Communication Engineering*.

[19] Bhatnagar G., Wu Q. M. J., Liu Z. (2013). Directive contrast based multimodal medical image fusion in NSCT domain. *IEEE Transactions on Multimedia*.

[20] Singh S., Gupta D., Anand R. S., Kumar V. (2015). Nonsubsampled shearlet based CT and MR medical image fusion using biologically inspired spiking neural network. *Biomedical Signal Processing and Control*.

[21] Liu X., Mei W., Du H., Bei J. (2016). A novel image fusion algorithm based on nonsubsampled shearlet transform and morphological component analysis. *Signal, Image and Video Processing*.

[22] Olshausen B. A., Field D. J. (1996). Emergence of simple-cell receptive field properties by learning a sparse code for natural images. *Nature*.

[23] Yang B., Li S. (2010). Multifocus image fusion and restoration with sparse representation. *IEEE Transactions on Instrumentation and Measurement*.

[24] Yang B., Li S. (2012). Pixel-level image fusion with simultaneous orthogonal matching pursuit. *Information Fusion*.

[25] Wang J., Peng J., Feng X., He G., Wu J., Yan K. (2013). Image fusion with nonsubsampled contourlet transform and sparse representation. *Journal of Electronic Imaging*.

[26] Liu Y., Liu S., Wang Z. (2015). A general framework for image fusion based on multi-scale transform and sparse representation. *Information Fusion*.

[27] Aharon M., Elad M., Bruckstein A. (2006). K-SVD: an algorithm for designing overcomplete dictionaries for sparse representation. *IEEE Transactions on Signal Processing*.

[28] Huang J., Huang X., Metaxas D. Learning with dynamic group sparsity.

[29] Deshmukh M., Bhosale U. (2010). Image fusion and image quality assessment of fused images. *International Journal of Image Processing*.

[30] Wang Z., Bovik A. C., Sheikh H. R., Simoncelli E. P. (2004). Image quality assessment: from error visibility to structural similarity. *IEEE Transactions on Image Processing*.

[31] Qu G. H., Zhang D. L., Yan P. F. (2002). Information measure for performance of image fusion. *IEEE Electronics Letters*.

[32] Aslantas V., Bendes E. (2015). A new image quality metric for image fusion: the sum of the correlations of differences. *AEÜ - International Journal of Electronics and Communications*.

[33] Wang Z., Bovik A. C. (2002). A universal image quality index. *IEEE Signal Processing Letters*.

[34] Piella G., Heijmans H. A new quality metric for image fusion.

[35] Xydeas C. S., Petrović V. (2000). Objective image fusion performance measure. *IEEE Electronics Letters*.

[36] Han Y., Cai Y., Cao Y., Xu X. (2013). A new image fusion performance metric based on visual information fidelity. *Information Fusion*.

